# The Microbiota-Gut-Brain Axis in Mental Health and Medication Response: Parsing Directionality and Causality

**DOI:** 10.1093/ijnp/pyaa088

**Published:** 2021-03-06

**Authors:** Thomaz F S Bastiaanssen, John F Cryan

**Affiliations:** Department of Anatomy and Neuroscience, School of Medicine, College of Medicine & Health, and APC Microbiome Ireland, University College Cork, Cork, Ireland

**Keywords:** Microbiota-gut-brain axis, psychotropics, microbiome

## Abstract

There is increasing evidence for the role of the microbiome in various mental health disorders. Moreover, there has been a growing understanding of the importance of the microbiome in mediating both the efficacy and side effects of various medications, including psychotropics. In this issue, Tomizawa and colleagues report on the effect of psychotropic drugs on the gut microbiome of 40 patients with depression and/or anxiety disorders. In their longitudinal cohort, the authors find that antipsychotics, but not anxiolytics, decrease microbiome alpha diversity. They further find that antipsychotics dosage was negatively correlated with alpha diversity in these patients. The health consequences of these microbiome alterations remain to be fully understood. In this commentary, we will discuss such findings through the lens of several recent studies on the microbiota-gut-brain axis. We also use the paper as a backdrop to discuss directionality and, by extension, causality in relation to microbiota-gut-brain-brain signaling.

## Introduction

The microbiome-gut-brain axis refers to the complex bi-directional system of communication that exists between the gut microbiome and the brain (Cryan et al., 2019). In recent years, it has become increasingly clear that the microbiota-gut-brain axis is involved in psychiatric disorders such as depression and anxiety disorders (Bastiaanssen et al., 2020; Cruz-Pereira et al., 2020). Indeed, a primer on the microbiome in the context of psychiatry was previously published in the “Making Sense of” series in this journal (Bastiaanssen et al., 2019). In this issue, Tomizawa and colleagues present an interesting study reinforcing the role of the microbiome in anxiety and depression (Tomizawa et al., 2020). In a cohort of patients with anxiety and depression, the authors report a decrease in microbial diversity and alterations in GABA and tryptophan metabolism in the microbiome after treatment with psychotropics, drugs that act on the central nervous system and are used in the treatment of psychiatric disorders (Cussotto et al., 2019).

**Figure 1.**
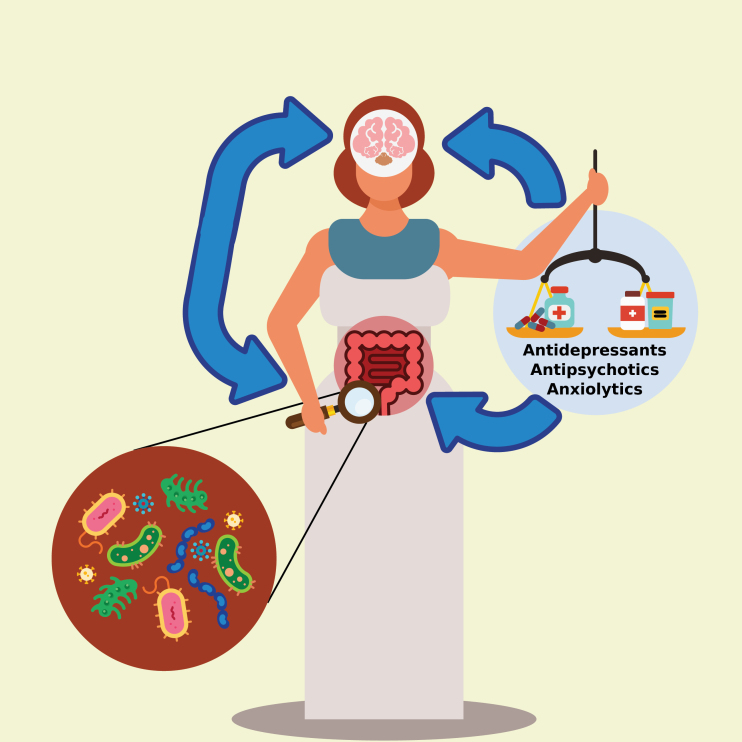
The microbiota-gut-brain axis is affected by psychotropic medication. The gut microbiota and the brain are in bi-directional communication, each influencing the other. External factors such as psychotropics can affect both the gut microbiota and the brain. Blue arrows represent the directionality of the effects.

Such studies are invaluable to the field, building on previous findings and moving the field forward. However, there are many questions that remain, and these are common to all areas of medicine that the microbiome is implicated in. Often, it remains unclear why the alterations of the microbiome are linked to the condition; the mechanism and directionality remain uncertain. Indeed, in the case of psychiatry, the microbiome is known to be in bi-directional communication with the brain, which can complicate teasing apart which organ is influencing which. Complicating matters further, it is often hard to rule out a hidden third factor that drives both the microbiome and the brain. Moreover, the answer should likely be sought in all three (Figure 1). In this commentary, we will discuss all 3 of these directionalities as well as consider how to disentangle these drivers in further research. Formulating ideas about directionality is an important step toward detecting causality (Sugihara et al., 2012), which we will also briefly touch on.

### On the Difficulty of Comparing Microbiome Studies

As the microbiome field is still relatively young, there is a distinct heterogeneity in methodology between studies in terms of both measuring the microbiome and in bioinformatics analysis (Goodrich et al., 2014; Clooney et al., 2016; McLaren et al., 2019). Recently, there have been calls to develop unified methodologies, though the rapidly developing nature of the field means that approaches are constantly in development as well, making it problematic to make strong prescriptive statements on methodology (Amos et al., 2020). An example of heterogeneity in methodology can be seen in the paper from Tomizawa and colleagues whereby they take advantage of the QIIME framework, which facilitates microbiome analysis in an easily standardizable and reproducible methodology (Caporaso et al., 2010). While QIIME is a fine framework for analysis, more recent frameworks, such as QIIME2, are available (Estaki et al., 2020). Similarly, the authors use the outdated Greengenes database, which is the default option in QIIME, when inferring function, but use the more up-to-date SILVA132 database for the rest of their analysis, thus introducing a level of incongruence in their study. Yet, despite directly comparing studies being problematic, numerous studies have elucidated parts of the microbiome-gut-brain axis directionality puzzle. There are at least 3 perspectives from which to view such analysis.

### Microbiome Influencing Brain

The first axis of directionality we will discuss is the gut microbiome influencing the brain. This would entail the microbiota directly or indirectly inducing a certain psychiatric condition. Recently, a Flemish group made an important step to elucidate directionality in the microbiota-gut-brain axis in a large cross-population cohort (Valles-Colomer et al., 2019). Not only did the group discover that participants with a lower psychological quality-of-life score were more likely to have a certain type of microbiome, they also found that this microbiome composition was associated with a different composition of gut-brain modules, functional modules representing functions that take place in the microbiome and are thought to influence the host brain, such as GABA or serotonin metabolism. Although this does not represent direct evidence that these modules are influencing the host, the fact that specific functions that are involved in host mood are altered is a good indication that the alterations in the microbiome are not random and rather likely have a specific effect on the host. The utility of such modules has now been employed across a variety of studies (Butler et al., 2020; Donoso et al., 2020; van de Wouw et al., 2020; Zhu et al., 2020).

Notably, Tomizawa and colleagues do not find broad functional differences between patients taking psychotropics and patients that were not in the current study, though they do report differences in the gut-brain modules in their supplementary data, including alterations in GABA and tryptophan metabolism. The question remains: Would we expect to observe a difference in the type of functional profile between a microbiome altered by a treatment “per se” and a microbiome that is perturbed in the disease and thus warrants the treatment (i.e., a dysbiotic microbiome)? Typically, we tend to consider decreased microbiome diversity to be detrimental to host health. However, as the authors rightly discuss, such generalizations are likely to be disease dependent and overly simplistic (Ma et al., 2019).

When discussing the potential of the microbiome to alter mood and behavior, we need to acknowledge the important role that animal models have played. Though there have been recent calls for more rigid and powerful methodological frameworks (Walter et al., 2020), several groups have reported being able to induce endophenotypes relevant to major depressive disorder (Kelly et al., 2016; Zheng et al., 2016) in rodents using fecal microbiota transplantation from patient donors. Indeed, the same group previously reported fecal microbiota transplantation in humans with irritable bowel syndrome improving psychiatric symptoms (Kurokawa et al., 2018).

### Brain Influencing Microbiome

It has often been reported that stress alters the microbiome (Foster et al., 2017), though this is best known in animals. There are substantial difficulties with designing human trials investigating the effect of brain state on the microbiome in the context of psychiatry as it would likely involve following participants since before the development of the disorder. Nevertheless, there have been some studies reporting changes in the microbiome after brain injury or stroke (Pathare et al., 2020).

Rhetorically, the possibility that the altered microbiome state could be compensatory has been suggested (Walter et al., 2020). Indeed, in an adjacent field, host genetics has been causally linked to an improved insulin response in the gut, protecting against type 2 diabetes (Sanna et al., 2019). It is thus not inconceivable that a host’s genetic factors that drive propensity towards psychological disorders would have an analogous effect on the microbiome, manifesting as a typical microbiome composition, caused by the disorder.

In the context of the current study, it seems a stretch to insinuate that a change in the patient psychiatric status because of treatment would result in a change in the microbiome directly. However, more longitudinal studies are required.

### A Third Driving Factor

Last, there is the scenario of a third factor driving changes in both the microbiome and the brain. In this study, the authors report changes in microbiome diversity after administration of psychotropic drugs. Recently, similar findings were also reported in a rodent study, where the administration of psychotropic drugs, specifically fluoxetine, lithium, valproate, and aripiprazole, altered the microbiome in a specific manner (Cussotto et al., 2018, 2019). In the rodent study, alterations in gastrointestinal function were also reported, though it remains unclear whether these effects were due to the altered microbiome or the psychotropic drugs. In a seminal in vitro study, the antimicrobial effects of common pharmaceuticals were assessed (Maier et al., 2018). It is conceivable that the drop in alpha diversity reported in the study in this issue is due to the antimicrobial activity of psychotropics. This begs the question whether this drop in alpha diversity affects the host and, if it does, how.

Analogously, in a longitudinal cohort of healthy volunteers following a diet rich in unpasteurized dairy products during a cookery course, both functional and compositional changes were reported. In addition, psychological measures showed improvement, though the cohort was healthy to begin with (Butler et al., 2020). Due to the observational nature of the cohort, it remains impossible to say whether these psychological effects were the product of the change in diet or microbiome or simply the anxiolytic effect of the course itself.

### Moving Forward

The concept of the microbiome-gut-brain axis has been established by a plurality of studies in recent years. Now, mechanism and directionality remain to be understood. Several promising studies have been published recently that represent a shift towards the question of directionality. Though unrelated to mental health, a recently published study following daily dietary intake and microbiome composition over 17 days convincingly shows that diet explains some degree of variance in the microbiome composition (Johnson et al., 2019). In the current study, Tomizawa and colleagues also use longitudinal sampling to strengthen their statistical framework. Following the success of longitudinal microbiome studies, statistical frameworks have been introduced to the field to better harness longitudinal microbiome data (Martino et al., 2020).

In certain cases, Mendelian randomization could be employed to infer causality. In short, Mendelian randomization is a statistical technique that typically leverages genetic information of the host, along with the fact that genetics largely remain fixed throughout lifespan, to make statements on causality and directionality between genetics and a phenotype (Sanna et al., 2019). When approaching causality in such a statistical manner, it is helpful to consider Granger causality, a special and useful case of causality that simply requires that knowledge of the occurrence *A* helps predict the occurrence of *B* to establish Granger causality (Granger, 1969).

Using a traditional experimental approach in the sense that it is rooted in Koch’s postulates (Koch, 1876), a group was able to establish causality by determining that a species of bacteria provided resistance against colitis by first detecting the candidate microbe bioinformatically and then testing the phenomenon in a follow-up experiment (Surana and Kasper, 2018). While sometimes impractical, these types of experiments arguably represent the most straightforward and robust tool to uncover causality.

The findings of Tomizawa and colleagues make an important contribution to the field, confirming that psychotropics alter the microbiome in a clinical cohort in terms of both diversity of the gut microbiome and its neuroactive potential. Yet, much work remains to be done. Teasing out which factors are driven by which part of the microbiota-gut-brain axis in psychopathology represents an important milestone in discovering the molecular mechanism and therefore formulating therapeutic targets. Considering recent findings while designing experiments will allow us to effectively pick up these driving factors, moving the field forward and improving treatment outcomes.

## Acknowledgments

None.

## Statement of Interest

Prof. Cryan is funded by Science Foundation Ireland SFI/12/RC/ 2273_P2, the Saks Kavanaugh Foundation, EU H2020 project DLV-848228 DIS-COvERIE, and Swiss National Science Foundation project CRSII5_186346/NMS2068. Prof. Cryan has received research funding from 4D Pharma, Cremo, Dupont, Mead Johnson, Nutricia, and Pharmavite; has been an invited speaker at meetings organized by Alimentary Health, Alkermes, Ordesa, and Yakult; and has served as a consultant for Alkermes and Nestle.
